# A Rail Fault Diagnosis Method Based on Quartic C^2^ Hermite Improved Empirical Mode Decomposition Algorithm

**DOI:** 10.3390/s19153300

**Published:** 2019-07-26

**Authors:** Hanzhong Liu, Chaoxuan Qin, Ming Liu

**Affiliations:** 1 School of Automation, Nanjing Institute of Technology, Nanjing 211167, China; 2 Department of Electronic and Computer Engineering, Hong Kong University of Science and Technology, Hong Kong, China; 3 School of Mechanical Engineering, Nanjing University of Science and Technology, Nanjing 210094, China

**Keywords:** empirical mode decomposition (EMD), fault diagnosis, Hermite interpolation, kurtosis, approximate entropy

## Abstract

For compound fault detection of high-speed rail vibration signals, which presents a difficult problem, an early fault diagnosis method of an improved empirical mode decomposition (EMD) algorithm based on quartic C^2^ Hermite interpolation is presented. First, the quartic C^2^ Hermite interpolation improved EMD algorithm is used to decompose the original signal, and the intrinsic mode function (IMF) components are obtained. Second, singular value decomposition for the IMF components is performed to determine the principal components of the signal. Then, the signal is reconstructed and the kurtosis and approximate entropy values are calculated as the eigenvalues of fault diagnosis. Finally, fault diagnosis is executed based on the support vector machine (SVM). This method is applied for the fault diagnosis of high-speed rails, and experimental results show that the method presented in this paper is superior to the traditional EMD algorithm and greatly improves the accuracy of fault diagnosis.

## 1. Introduction

High-speed trains have developed rapidly in the recent years. Compared to the traditional trains, high-speed trains have advantages such as fast speed, strong carrying capacity, green environmental protection, high safety, and good economic benefits. However, when the high-speed train accelerates and brakes, the rails are prone to severe friction, crushing, and impact. Under harsh working conditions, the rails easily fail. If these failures are not discovered in time, the defects in the rails can expand rapidly causing the rails to break, which can result in serious safety concerns such as accidents. Hence great importance is placed on early fault diagnosis studies of the high-speed rails.

Domestic and foreign scholars have conducted a lot of research on the safety of high-speed rails. Yin Kai used image detection and other related technologies to detect faults in the rails [1]. Li Haiqing analyzed high-speed rail vibration signals and used an empirical mode decomposition (EMD) algorithm to extract the rail damage information for fault detection [2]. Xu Peng et al. used the differential eddy current detection system to detect cracks in high-speed rails and obtained the amplitude and phase changes from the crack detection signals; then they deduced the relation between the detection signal and the crack [3]. Xiongxiang Liu used deep convolutional neural networks to identify the surface defects in the two-dimensional images of the rails [4]. However, the deep learning model has high computational complexity and limited recognition accuracy, which may lead to misjudgment. There are other nondestructive testing methods such as magnetic particle testing [5], ray detection [6], and ultrasonic testing [7]. Ultrasonic testing is widely used among them, as its accuracy is high and it is easily operated. However, for surface micro-cracks, the detection effect is often affected by the rail surface roughness. At present, the commonly used flaw detection vehicles can only detect the defects on the rail surface, it is difficult to detect damage inside the rail, as internal damage is usually extremely serious.

Common faults in rails include damage at the inner rail head, rail surface wear, damage at the rail waist or bottom [2]. Because of the overload and strong impact during the train running, the fault mode includes various damage, which may be a single fault mode or a combination of fault modes. When the rail is in a fault state, its vibration signal will contain abnormal components such as impact and friction. By analyzing the vibration signal, the fault features of the rail can be obtained at an early stage.

Many research about the fault features extraction from vibration signals and diagnosis have been carried out over the years. Algorithms based on the Fourier transform, such as short-time Fourier transforms, Winger−Ville, and wavelet transforms [8,9,10,11], have played important roles, but the performances of these algorithms is limited due to their lack of adaptability when dealing with nonlinear nonstationary signals. Huang presented the EMD algorithm, which is a signal processing method based on the instantaneous frequency [12]. It has strong adaptability to non-stationary signals and has been widely used. However, the traditional EMD algorithm has problems such as curves fitting with the overshoot, undershoot, endpoint swing, and mode mixing. Therefore, the research of EMD focuses on the suppression of the mode mixing and optimization of the sifting procedure. For mode mixing, scholars usually blame the existence of the intermittent signal, as the frequency components of the signal are too close and the amplitude of the low-frequency signal is too large [13,14]. The noise-assistant methods are widely used to settle the mode mixing problem. Wu and Huang [15] added white Gaussian noises to the original signal to strengthen the high frequency component, which is called Ensemble Empirical Mode Decomposition (EEMD) method. Based on the EEMD, the complementary ensemble empirical mode decomposition method (CEEMD) [16] and complete EEMD with adaptive noise method (CEEMDAN) [17] are also the prominent methods to solve the mode mixing problem. In recent years, extensive efforts have been carried out for mode mixing prevention [18,19,20,21]. For optimization of sifting procedure, Yang used the B-spline interpolation method to improve the EMD algorithm; though, the signal length was assumed to be infinite and the endpoint effect was introduced [22]. Zhu W used the piecewise cubic Hermite interpolation method to improve the envelope process of the EMD algorithm and obtained good results [23]; however, the curve was bent artificially so that its smoothness was affected. Di Z et al. used the cubic trigonometric cardinal interpolation method with adjustable shape to carry out EMD algorithm, accompanied with great complexity [24].

In this paper, the rail vibration signal is decomposed with the quartic C^2^ Hermite interpolation method improved EMD algorithm, and the intrinsic mode function (IMF) components are obtained. Additionally, singular value decomposition [25] (SVD) for the IMF components is performed to determine the principal components of the signal. Then, through the cross correlation coefficient calculation between the IMF components and the original signal, the IMF components with larger correlation coefficients are reconstructed. Then, kurtosis and approximate entropy values of the reconstructed signal are calculated as fault eigenvalues, and finally, early faults of rails are detected and identified by support vector machine [26] (SVM) classifier based on eigenvalues.

The contributions of the paper are as follows:

(1) In order to solve the undershoot problem caused by traditional EMD algorithm, a quartic C^2^ Hermite interpolation algorithm is proposed to make the curve more smooth and flexible.
(2) Adopt a low-cost and simple method of rail fault diagnosis, use the acceleration sensor to obtain the rail vibration signal, and analyze the signal so as to carry out effective fault diagnosis.
(3) Use quartic C^2^ Hermite improved-EMD algorithm to analyze the rail vibration signal and extract the kurtosis and approximate entropy for fault diagnosis. Experiments show that this method can diagnose the fault effectively.

The rest of the paper is arranged as follows. Section 2 introduced the traditional EMD algorithm and its undershoot problem. Aiming at addressing this problem, an improved EMD algorithm with quartic C^2^ Hermite [27] interpolation is presented. Section 3 introduced the singular value decomposition algorithm and the signal reconstruction method used in this paper. Section 4 introduced the fault features used in this paper, which are kurtosis and approximate entropy, and the whole rail fault diagnosis process. Section 5 introduced relevant experiments to verify the effectiveness of the algorithm.

## 2. Empirical Mode Decomposition

### 2.1. EMD Principle

The EMD method was proposed by Huang et al. in 1988. This method exhibits outstanding performance in dealing with non-linear and non-stationary random signals. Therefore, it is widely used in the fields of signal denoising, signal features extraction, and so on. The EMD method decomposes the original signal into multiple IMF components based on the local feature time scale [12]. The conditions that must be met for IMF are as follows:

(1) The numbers of extreme points and zero points must be equal or at most different in one over the length of the data;
(2) At any data point, the average of the envelope of the local maximum and that of the local minimum must be zero.

The signal that can be decomposed with the EMD algorithm needs to satisfy the following conditions:

(1) Have at least two extreme points, including a maximum point and a minimum point;
(2) A signal feature time scale is determined by the time interval between extreme points;
(3) Have no extreme points; the first-order or higher-order derivatives of inflection points can be taken as extreme points.

For a signal named X, the EMD steps are as follows:

(1) Determine all the local extreme points of the signal, use the cubic spline interpolation to fit all local maximum points to form the upper envelope curve, and fit all local minimum points to form the lower envelope curve.
(2) Calculate $h_{0}\left(t\right)=x\left(t\right)-\frac{x_{0}^{u}\left(t\right)+x_{0}^{d}\left(t\right)}{2}$.
(3) Determine whether $h_{0}\left(t\right)$ satisfies the conditions of the IMF. If they are satisfied, then $h_{0}\left(t\right)$ is the first IMF component; otherwise, repeat steps 1 and 2 for $h_{0}\left(t\right)$ until the IMF conditions are satisfied. The IMF component after the first filtration is recorded as $s_{1}\left(t\right)$.
(4) Calculate the remaining signal $r_{1}\left(t\right)=x\left(t\right)-s_{1}\left(t\right)$, repeat step 1 to 3 for $r_{1}\left(t\right)$ to get the second IMF component, and in this way, repeat *n* times loop to get *n* IMF components. Terminate the step when the remainder is a monotonic signal or meets a given condition.

### 2.2. Undershoot

**Figure 1:**
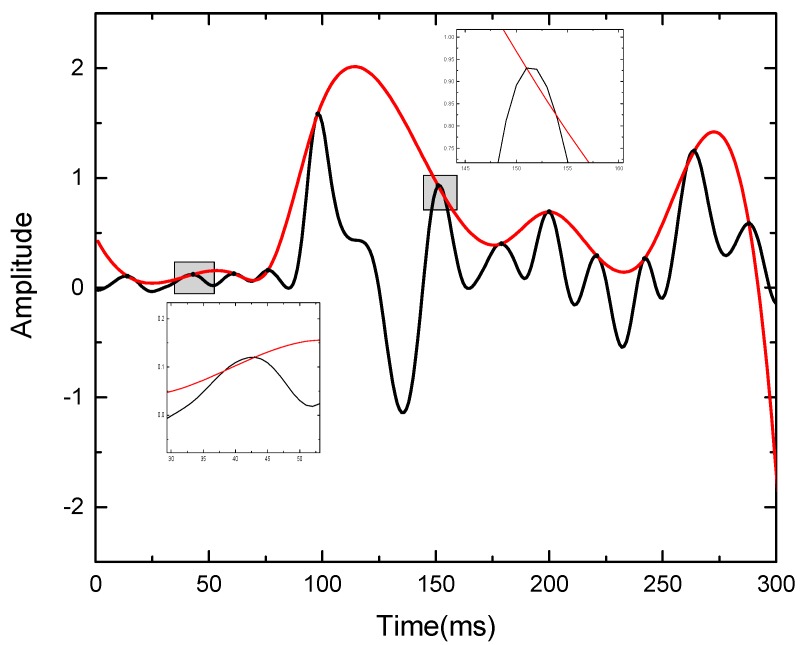
Undershoot phenomenon.

The signal filtration process is the most important part of the EMD algorithm and directly affects the decomposition results. Traditionally, when the cubic spline curve is used to fit extreme points to form the envelope curve, the undershoot phenomenon easily occurs. As shown in Figure 1, the upper envelope curve is fitted by cubic spline interpolation. The ideal upper envelope curve should satisfy $x^{c}\left(i\right)\geq x\left(i\right),\forall i=1,2,\cdots ,N$ anywhere. However, there are two places where the values of the envelope curve are less than those of the original signal, and this phenomenon is called “undershoot”. The envelope curve cannot surround the original signal curve completely, resulting in meaningless IMF components during the decomposition process, or signal distortion during the signal reconstruction process.

The reason for the undershoot phenomenon is that the traditional fitting algorithm with cubic spline interpolation only satisfies C^1^ continuous, such that the shape is fixed under the given conditions and lacks flexibility. So the fitting performance is poor when the non-linear and non-stationary signal changes greatly.

### 2.3. Improved EMD Algorithm

In order to solve the drawbacks of the method with cubic spline interpolation, the trigonometric interpolation method was used to fit the envelope curve [24]. Yang proposed a method with piecewise cubic spline interpolation; however, the method has poor continuity and only satisfies C^1^ continuous [22]. Oberlin T proposed a method with which the envelope curve can be constructed directly to replace the method with cubic spline interpolation [28]; Zhu Weifang used a method with piecewise cubic Hermite interpolation to fit the envelope curve [23], which mainly used Lagrangian method to get the minimum value and optimized the derivative value at extreme points. However, this method also does not satisfy C^2^ continuous. Some common interpolation methods and their pros and cons are shown in Table 1.

In this paper, the EMD algorithm is improved by the quartic C^2^ Hermite interpolation [27]. Suppose there are *n +* 1 nodes in the interval [a,b], which satisfy $x_{0}<x_{1}<\cdots <x_{n}$, then the traditional standard cubic Hermite basis function is

(1) $$\{\begin{array}{l} a_{i}\left(t\right)=1-3t^{2}+2t^{3}\\ a_{i+1}\left(t\right)=3t^{2}-2t^{3}\\ b_{i}\left(t\right)=t-2t^{2}+t^{3}\\ b_{i+1}\left(t\right)=-t^{2}+t^{3} \end{array}$$

where t=x−xixi+1−xi.

This is convenient for the calculation with the standard cubic Hermite interpolation method, which is a common method used in engineering. However, the shape of the fitting curve obtained from this method is fixed, which lacks adaptability and only satisfies C^1^ continuous. In this paper, a quartic C^2^ Hermite interpolation method is presented, which adds a control factor to adjust the shape of the curve. The basis function is as follows:

(2) $$\{\begin{array}{l} F_{i}\left(t\right)=\lambda_{i}t^{4}-2\left(\lambda_{i}-1\right)t^{3}+\left(\lambda_{i}-3\right)t^{2}+1\\ F_{i+1}\left(t\right)=-\lambda_{i}t^{4}+2\left(\lambda_{i}-1\right)t^{3}-\left(\lambda_{i}-3\right)t^{2}\\ G_{i}\left(t\right)=\lambda_{i}t^{4}-\left(2\lambda_{i}-1\right)t^{3}+\left(\lambda_{i}-3\right)t^{2}+t\\ G_{i+1}\left(t\right)=-\lambda_{i}t^{4}+\left(2\lambda_{i}-1\right)t^{3}-\left(\lambda_{i}-3\right)t^{2} \end{array}$$

where λ is a control factor to adjust the shape of the curve.

The basis function satisfies such conditions as follows:

(3) $$\left\{ \begin{array}{l} F_{i}\left(0\right)=F_{i+1}\left(1\right)=1\\ F_{i}\left(1\right)=F_{i+1}\left(0\right)=0;\\ F_{i}^{\prime }\left(0\right)=F_{i}^{\prime }\left(1\right)=F_{i+1}^{\prime }\left(0\right)=F_{i+1}^{\prime }\left(1\right)=0\\ G_{i}\left(1\right)=G_{i}\left(1\right)=G_{i+1}\left(0\right)=G_{i+1}\left(1\right)=0\\ G_{i}^{\prime }\left(0\right)=G_{i+1}^{\prime }\left(1\right)=1\\ G_{i}^{\prime }\left(1\right)=G_{i+1}^{\prime }\left(0\right)=0\\ F_{i}\left(t\right)+F_{i+1}\left(t\right)=1 \end{array}\right.$$

The method with quartic Hermite interpolation has the same properties as the one with traditional cubic Hermite interpolation, but the shape of the curve is more flexible due to the parameter λ. When the parameter λ equals 0, the curve is standard cubic Hermite spline curve, which satisfies $C^{1}$ continuous. The quartic Hermite spline curve at the interval of [a,b] is

(4) $$C_{i}\left(x\right)=F_{i}\left(t\right)y_{i}+F_{i+1}\left(t\right)y_{i+1}+{\dot{y}}_{i}G_{i}\left(t\right)\Delta x_{i}+{\dot{y}}_{i+1}G_{i+1}\left(t\right)\Delta x_{i},i=0,1,\cdots ,n-1$$

where $y_{i}$ is the function value of curve, ${\dot{y}}_{i}$ is its first derivative value, and $\Delta x_{i}=x_{i+1}-x_{i}$. If the curve satisfies C^2^ continuous, the second derivative must satisfy the condition as follows:

(5) $$C^{\prime \prime }\left(x-0\right)=C^{\prime \prime }\left(x+0\right)$$

And the continuous equation by calculation is as follows

(6) $$\begin{array}{l} \lambda_{i}\left(\Delta y_{i}+\Delta x_{i}\Delta {\dot{y}}_{i}\right)\Delta x^{2}{}_{i+1}-\lambda_{i+1}\left(\Delta y_{i+1}+\Delta x_{i+1}\Delta {\dot{y}}_{i+1}\right)\Delta x^{2}{}_{i}\\ =-\Delta x^{2}{}_{i+1}\left(3\Delta y_{i}+\Delta x_{i}\left({\dot{y}}_{i}+2{\dot{y}}_{i+1}\right)\right)-\Delta x^{2}{}_{i}\left[3\Delta y_{i+1}+\Delta x_{i+1}\left({\dot{y}}_{i+2}+2{\dot{y}}_{i+1}\right)\right] \end{array}$$

When λ satisfies the constraint condition as in Equation (6), the plotted curve will satisfy C^2^ continuous and it will be smoother.

### 2.4. The Shape of the Envelope Curve Determination

It is known from Section 2.3 that the shape of the fitting curve with quartic C^2^ Hermite interpolation is controlled by the parameter λ, and the curve cluster can be obtained with a different parameter λ. This paper draws the idea from reference [23] to find the curve whose length is the shortest in the curve cluster formed by different λ. Generally speaking, excessive length of curve will cause fitting overshoot. The curve length can be expressed as

(7) $$L=\sum_{i=1}^{n-1}\int_{x_{i}}^{x_{i+1}}\sqrt{1+\dot{C}{\left(t\right)}^{2}}dt,i=1,2,\cdots n-1$$

The conditions for searching the optimal curve are as follows:

(8) $$\begin{array}{l} \min L\\ st.\left\{ \begin{array}{l} 0<\lambda <2\\ L=\sum_{i=1}^{n-1}\int_{x_{i}}^{x_{i+1}}\sqrt{1+\dot{C}{\left(t\right)}^{2}}dt,i=1,2,\cdots n-1\\ C_{i}\left(x\right)=F_{i}\left(t\right)y_{i}+F_{i+1}\left(t\right)y_{i+1}+{\dot{y}}_{i}G_{i}\left(t\right)\Delta x_{i}+{\dot{y}}_{i+1}G_{i+1}\left(t\right)\Delta x_{i},i=0,1,\cdots ,n-1\\ \lambda_{i}\left(\Delta y_{i}+\Delta x_{i}\Delta {\dot{y}}_{i}\right)\Delta x^{2}{}_{i+1}-\lambda_{i+1}\left(\Delta y_{i+1}+\Delta x_{i+1}\Delta {\dot{y}}_{i+1}\right)\Delta x^{2}{}_{i}\\ =-\Delta x^{2}{}_{i+1}\left(3\Delta y_{i}+\Delta x_{i}\left({\dot{y}}_{i}+2{\dot{y}}_{i+1}\right)\right)-\Delta x^{2}{}_{i}\left[3\Delta y_{i+1}+\Delta x_{i+1}\left({\dot{y}}_{i+2}+2{\dot{y}}_{i+1}\right)\right] \end{array}\right. \end{array}$$

After many experiments, it is found that the optimal curves will appear only when λ<2. If λ is too large, the smoothness of the curve will be affected. The shape of the curve will be determined by using the genetic algorithm to find the optimal value of λ. The detailed steps are as follows.

(1) Code the variables λ∈(0,2], use binary coding, and divide the interval into 1024 subintervals.
(2) Calculate the fitness value of each individual according to Equation (8).
(3) Selection operator, elite individual preservation strategy and rotation method selection operator are used to calculate the probability of each selected individual and the cumulative probability in the whole population fitness Pi=fitness(i)∑populationfitness(i), $Q_{i}=\sum_{j=1}^{i}P_{j}$.
(4) Set the crossover probability $p_{c}$, and generates new individuals according to the single-point crossover rule.
(5) Set the mutation probability $p_{m}$. The mutation individual is determined randomly and the reverse mutation operator is used for mutation operation.
(6) Repeat steps (2)~(5) until the termination condition is reached.

## 3. Singular Value Decomposition and Signal Reconstruction

The matrix singular value is usually used for signal feature extraction because of its good stability [20]. The signal matrix named $X_{m\times n}$ is decomposed as follows:

(9) $$\begin{array}{l} X=U\Sigma V^{T}\\ =\left[u_{1},u_{2},\cdots ,u_{n}\right]\left[\begin{array}{cccc} \delta_{1} & & & \\ & \delta_{2} & & \\ & & \cdots & \\ & & & \delta_{n} \end{array}\right]\left[v_{,},v_{2},\cdots ,v_{n}\right] \end{array}$$

where U is matrix of left singular vectors and V is matrix of right singular vectors. $\Sigma =diag\left(\delta_{1},\delta_{2},\cdots ,\delta_{n}\right)$ is a singular value matrix and satisfies $\delta_{1}\geq \delta_{2}\geq \cdot \cdot \cdot \geq \delta_{n}$.

The percentage of signal components is calculated as

(10) $$\lambda K=\frac{\sum_{i=1}^{k}\delta_{i}}{\sum_{j=1}^{N}\delta_{j}},k=1,2\cdots N$$

where $\lambda_{k}$ represents the percentage of the first *k* signal components, and *N* is the number of IMF components obtained by EMD. The threshold value of percentage is set to 0.9, which means 90% of the original signal can be obtained. The IMF components are reordered according to the correlation coefficient calculated from each IMF and the original signal X. When $\lambda_{k}>0.9$, the first *k* IMF components are used for signal reconstruction, and the remaining *N−k* IMF components are abandoned. The cross-correlation coefficient can be expressed as follows:

(11) $$\gamma \left(X\left(t\right),IMFi\right)=\frac{\left|\mathrm{cov}\left(X\left(t\right),IMFi\right)\right|}{\sqrt{\mathrm{var}\left(X\left(t\right),X\left(t\right)\right)•\mathrm{var}\left(IMFj,IMFi\right)}}$$

where cov () represents the covariance function, var () represents the variance function, X(t) is the original signal, and $IMF_{i}$ represents the *i*th IMF component obtained by EMD. The range of the cross-correlation coefficient is [0,1], which reflects the matching degree between these two signals. The closer the value is to 1, the closer the component is to the original signal.

## 4. Fault Diagnosis Method

### 4.1. Fault Feature

Kurtosis is a dimensionless parameter that reflects the characteristics of signal distribution

(12) $$K=\frac{E{\left(x-\mu \right)}^{4}}{\delta^{4}}$$

where x is the signal to be analyzed, μ is the signal mean value, and δ is the signal standard deviation. The fault rail vibration signals are usually accompanied by impact components to which the kurtosis is sensitive [31], so the larger the kurtosis value is, the higher the proportion of impact components in the signal, therefore the kurtosis can reflect the rail fault.

Approximate entropy can measure the complexity of the signal [32]. The more complex the signal mode is, the more irregular the signal, and the larger the approximate entropy. The approximate entropy calculation method is as follows.

(1) The original signal is called X, which is an m-dimensional vector composed of continuous points on a time scale
  (13) $$X_{i}=\left[x\left(i\right),x\left(i+1\right),\cdots ,x\left(i+m-1\right)\right],i=1,\cdots ,N-m+1$$
(2) Calculate vector distance
  (14) $$d\left[X\left(i\right),X\left(j\right)\right]=\underset{k=0,\cdots ,m-1}{\max}\left(\left|x\left(i+k\right)-x\left(j+k\right)\right|\right)$$
(3) Given a threshold of similar tolerance represented by variable *r*, calculate each vector distance and get the number of less than r, which is expressed as $C_{i}^{m}\left(r\right)$,
  (15) Cim(r)=1N−mCont(d[X(i),X(j)]<r)i,j=1,⋯,N−m+1,i≠j
(4) Calculate the log values of $C_{i}^{m}\left(r\right)$ and get their mean value expressed as $\phi^{m}\left(r\right)$,
  (16) ϕm(r)=1N−m+1∑i=1N−m+1lnCim(r)
(5) Calculate the approximate entropy,
  (17) $$ApEn\left(m,r\right)=\underset{N\to \infty }{\lim}\left(\phi^{m}\left(r\right)-\phi^{m+1}\left(r\right)\right)$$

### 4.2. Fault Diagnosis Steps

**Figure 2:**
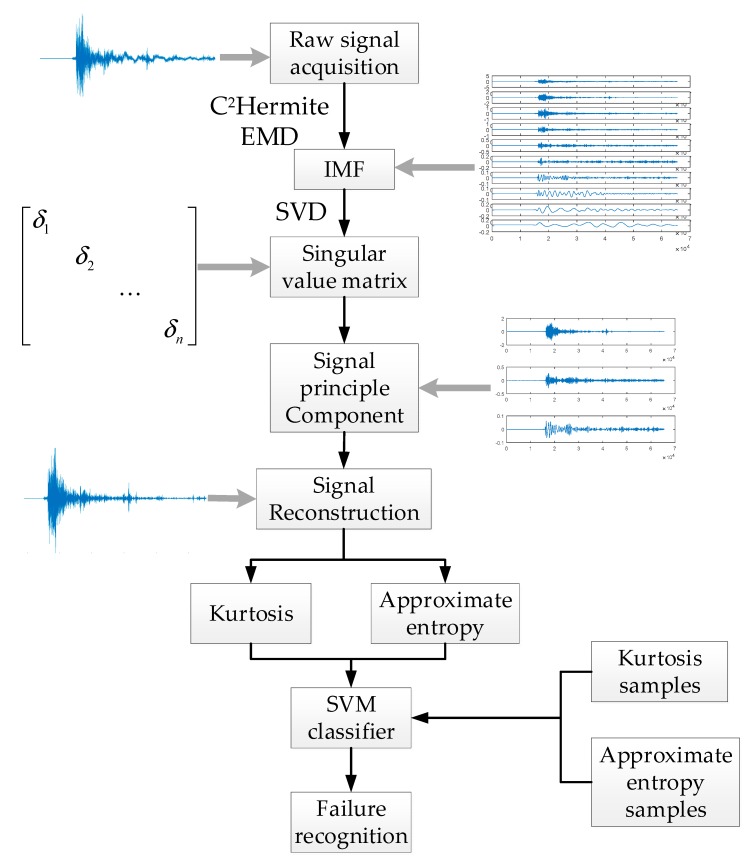
Flow chart of fault diagnosis.

The fault diagnosis steps are as follows and the flow chart is shown in Figure 2.

(1) Use an acceleration sensor to obtain the original vibration signal;
(2) Process the vibration signal with the EMD algorithm based on quartic C^2^ Hermite interpolation described in Section 2.3 and Section 2.4 to obtain the IMF components;
(3) Process the acquired IMF components by using the SVD algorithm to obtain a singular value matrix;
(4) Determine the number of primary components of the signal and reconstruct the signal;
(5) Calculate the kurtosis and approximate entropy of the reconstructed signal as the eigenvalues of fault diagnosis;
(6) Train the SVM classifier with kurtosis and approximate entropy values of training samples;
(7) Classify by the SVM classifier based on the different eigenvalues.

## 5. Experiment

### 5.1. EMD Envelope Experiment

**Figure 3:**
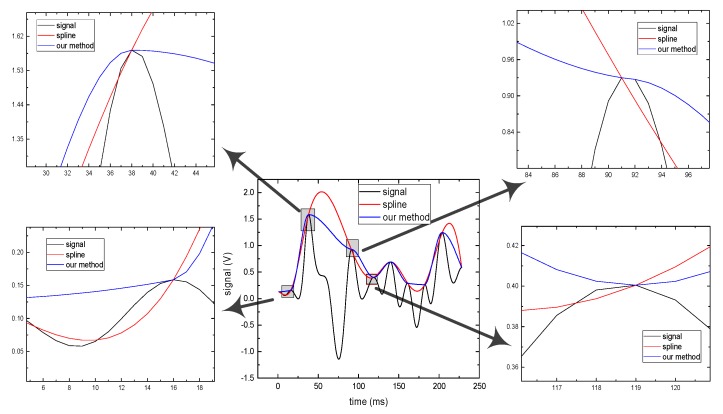
Envelope curve fitting of vibration signal.

The algorithms with quartic C^2^ Hermite interpolation and cubic Hermite interpolation are applied to the vibration signal processing. Figure 3 shows a part of the vibration signal, where the black curve is the original signal. The red is the upper envelope curve fitted with cubic Hermite interpolation algorithm and the blue is the upper envelope curve with the quartic C^2^ Hermite interpolation algorithm. It can be seen from Figure 3 that obvious overshoot will appear with the cubic Hermite interpolation algorithm during non-stationary signal processing, and undershoot phenomenon appear in many positions (shown in the zoom Figure 3). But the quartic C^2^ Hermite interpolation algorithm presented in this paper effectively solves the overshoot and undershoot problem.

### 5.2. EMD Simulation Experiment

The signal in the simulation experiment is as follows:

(18) s=0.7sin(2π×10t)+2sin(2π×100t)+(10+sin(2π×20t))cos(2π×50t)+noise

**Figure 4:**
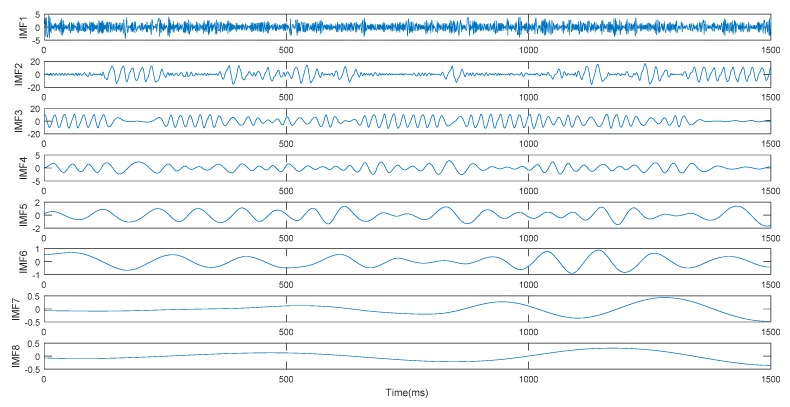
Intrinsic mode function (IMF) components obtained by the empirical mode decomposition (EMD) based on the C^2^ Hermite interpolation algorithm.

**Figure 5:**
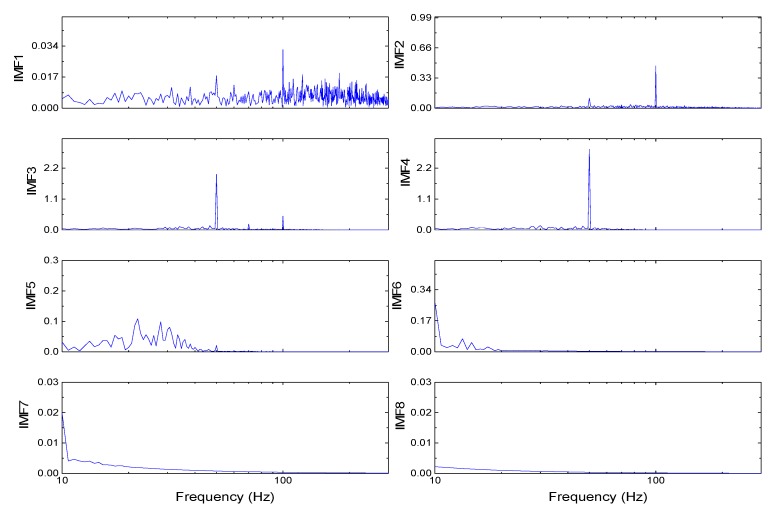
Components spectrograms obtained by EMD based on the C^2^ Hermite interpolation algorithm.

The simulation signal is composed of AM–FM and Gaussian white noise. The simulation platform is MATLAB2017a and the sampling frequency is set to 1000 Hz. In the simulation, the signal is processed separately with the traditional cubic Hermite interpolation algorithm, piecewise cubic Hermite interpolation algorithm, and the quartic C^2^ Hermite interpolation algorithm, which are presented in this paper. The IMF components obtained by EMD based on the quartic C^2^ Hermite interpolation algorithm and the corresponding spectrograms are shown in Figure 4 and Figure 5, respectively. As can be seen from the figure, the frequency of the first IMF component (IMF1) is distributed in each frequency band, and the extracted IMF1 component approximates Gaussian white noise. The spectrums of IMF2 and IMF3 are concentrated at 100 Hz and 50 Hz, while the spectrum of IMF5 is concentrated at 10 Hz. Therefore, every mode of the signal can be effectively extracted with the proposed algorithm.

Mode mixing may appear in the decomposition [12]. Mode mixing means that an IMF component contains extremely different feature time scales or similar feature time scales distributed in different IMF components, which is caused by uneven distribution of signal extremums, signal interruption, or pulse, etc. The common method to solve mode mixing is to add white noise to the signal to suppress abnormal signal [15,17].

Index of orthogonality (IO) and index of energy conservation (IEC) are important indexes for evaluating the EMD results. The expressions are presented in Equations (19) and (20). The IO shows the orthogonality of the IMF component obtained with the EMD algorithm. The closer the value is to 0, the better the orthogonality between the IMF components. The IEC shows the energy conservation after signal decomposition. The closer the value is to 1, the higher the energy conservation by EMD.

(19) $$\mathrm{IO}=\sum_{t=0}^{T}\left(\frac{\sum_{i=1}^{N}\sum_{j=1}^{N}f_{i}\left(t\right)f_{j}\left(t\right)}{X^{2}\left(t\right)}\right)$$

(20) $$\mathrm{IEC}=\sum_{t=0}^{}\frac{\sum_{i=1}^{n}{\left|f_{i}\left(t\right)\right|}^{2}}{{\left|X\left(t\right)-r\left(t\right)\right|}^{2}}$$

where X(t) represents the original signal, $f_{i}\left(t\right)$ and $f_{j}\left(t\right)$ represent the *i*-th and *j*-th IMF components by EMD respectively, and r(t) is a trend item.

Objective evaluation indicators are used to validate the algorithm presented in this paper, the result is shown in Table 2. The IO and IEC obtained with the algorithm presented in this paper are better than those obtained with the traditional cubic Hermite interpolation algorithm and piecewise cubic Hermite interpolation algorithm.

### 5.3. The Effect of Noise on the Quartic C^2^ Hermite Improved Empirical Mode Decomposition Algorithm

The influence of noise on the performance of EMD algorithm is analyzed through simulation experiments, and the simulation signal is:

(21) s=sin(2π×20t)+sin(2π×100t)+noise

**Figure 6:**
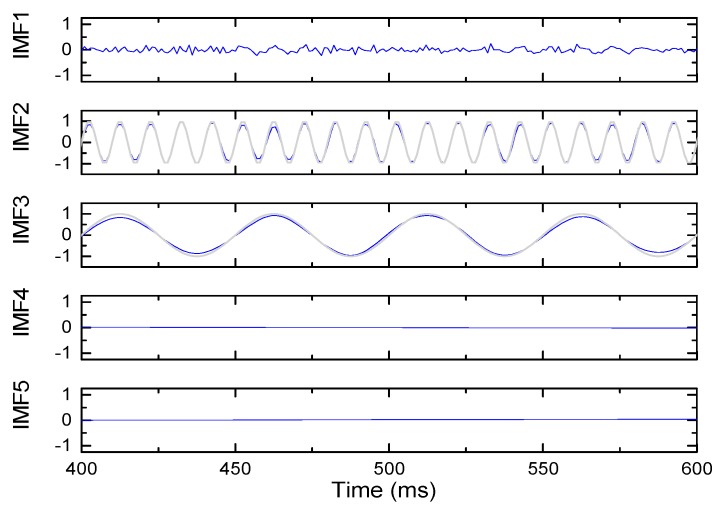
Components obtained by EMD based on the C^2^ Hermite interpolation algorithm.

The signal is composed of sinusoidal signal with frequency of 20 Hz and 100 Hz superimposed with noise signal. The sampling frequency is set as 1000 Hz, the signal length is 1 s, and the noise variance is 0–1. Under different noises, the algorithm proposed in this paper is used to process the signal respectively. Figure 6 shows the IMF component with noise variance of 0.01, IMF2 is approximately a sinusoidal signal of 100 Hz, and IMF3 is approximately a sinusoidal signal of 20 Hz. The blue lines in the figure are IMF decomposed by EMD algorithm, and the gray lines are sinusoidal signals of 100 Hz and 20 Hz respectively. It can be seen from the figure that the algorithm in this paper can effectively decompose the signal and extract typical components of the signal.

**Figure 7:**
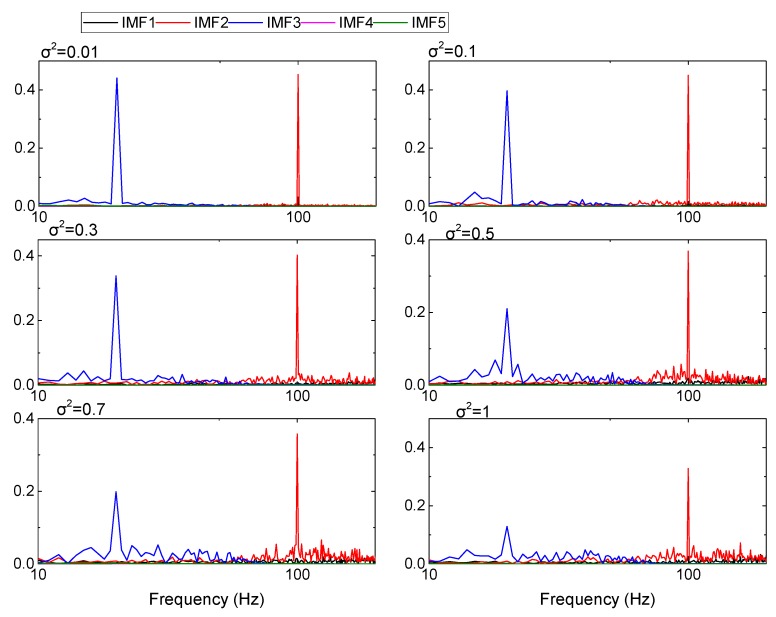
Signal decomposition result by EMD based on the C^2^ Hermite interpolation algorithm under different noise variances.

Figure 7 shows the spectrum diagrams of each IMF component decomposed when the variances are 0.01, 0.1, 0.3, 0.5, 0.7, and 1 respectively, and the abscissa is represented by log 10. As can be seen from the figure, the increase of noise has a certain impact on the EMD decomposition and has a more significant impact on the high-frequency region. However, signal frequency characteristics are not affected, even when the noise variance is large.

**Figure 8:**
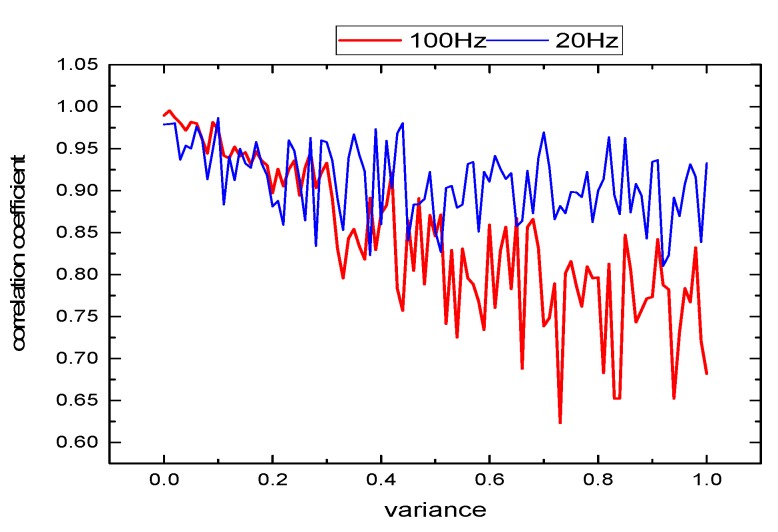
Correlation coefficient of two components under different noise variances.

The noise variance is increased from 0 to 1 with an interval of 0.01, and the EMD decomposition is carried out and the correlation coefficient is calculated. As shown in Figure 8, the red line is the correlation coefficient of IMF2 and 100 Hz sinusoidal signal, and the blue line is the correlation coefficient of IMF3 and 20 Hz sinusoidal signal. It can be seen that, with the increase of noise variance, the correlation coefficients of both components show a decreasing trend, and the reduction range of 20 Hz signal is significantly smaller than that of the 100 Hz signal. Since EMD algorithm has this characteristic, EMD algorithm can be used for signal noise reduction. In particular, when the target signal is distributed in the low-frequency component, the denoising effect is good. If the target signal frequency is high, the denoising effect is generally poor.

### 5.4. Fault Eigenvalues Extracted from Rail Vibration Signal

**Figure 9:**
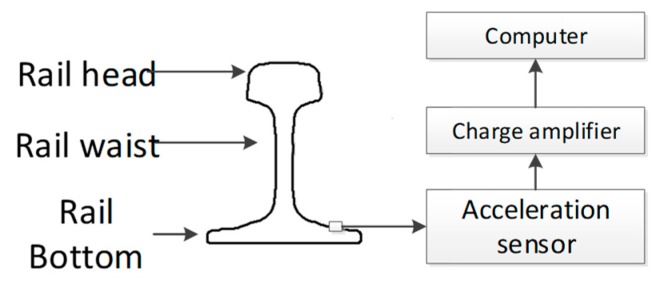
Composition of test System.

**Figure 10:**
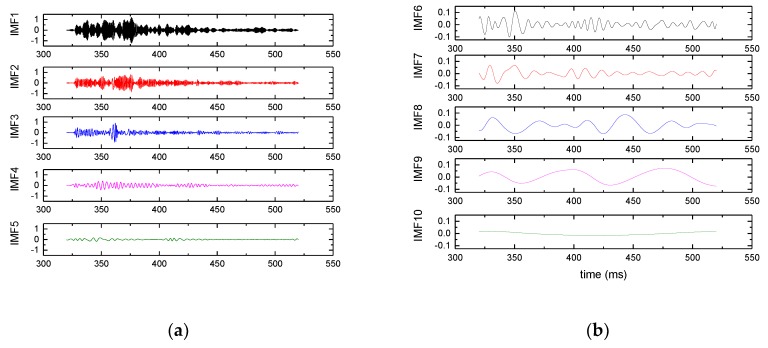
IMF components obtained by EMD. (**a**) IMF 1 to IMF 5, (**b**) IMF 6 to IMF 10.

The proposed algorithm is applied to vibration signal analysis to validate its effectiveness. The composition of the test system is shown in Figure 9, the rail is made with 60 kg/m standard steel. The sensors are mounted on the bottom of the rail so that the effect on train operation and track mechanical strength is minimal. The sensor used in the experiment is CYD103 piezoelectric acceleration sensor, its sensitivity is 20 pC/g. The sensor is connected to the YE5853 charge amplifier, which is used to modulate and transform the signals collected by the acceleration sensor for display on the computer and subsequent processing. The signal sampling frequency is 50 kHz. The IMF components of the signal obtained by EMD is as shown in Figure 10.

Singular value decomposition is performed for the IMF components obtained; the results are shown in Table 3. Suppose the accumulated percentage of principal components is 90%, then it can be seen that the percentage of the first eight principal component reaches 92.24%. So the signal can be reconstructed by using the first eight IMF components.

**Figure 11:**
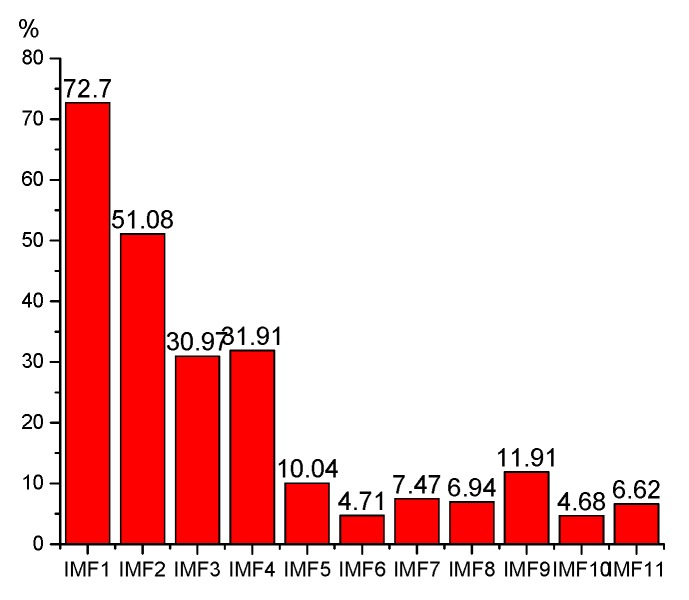
Cross correlation for the IMF components and the original signal.

As is shown in Figure 11, the cross correlation coefficient calculations between the IMF components and the original signal are performed. The first eight IMF components with high cross correlation coefficient are used for reconstruction.

**Figure 12:**
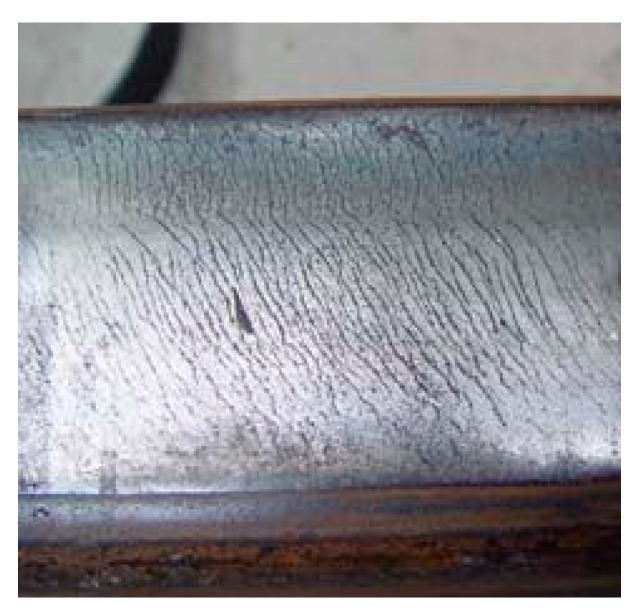
Rail surface fault.

According to a large number of investigations, the main failure forms of high-speed rails in China are rail surface peeling and corrugation, which is shown in Figure 12.

- Rail surface peeling damage

During train operation, due to the large braking, sliding friction is produced by the locked wheels on the rail surface, causing the local temperature to rise rapidly. When the rail cools, the raw surface of the hard and brittle metal gets damaged, resulting in the rail surface peeling phenomenon due to the large cyclical impact between the wheel and rail.

**Figure 13:**
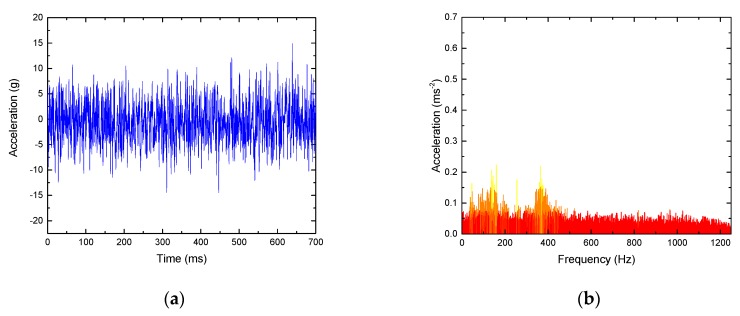
Analysis of a normal rail vibration signal. (**a**) Waveform; (**b**) frequency spectrum.

**Figure 14:**
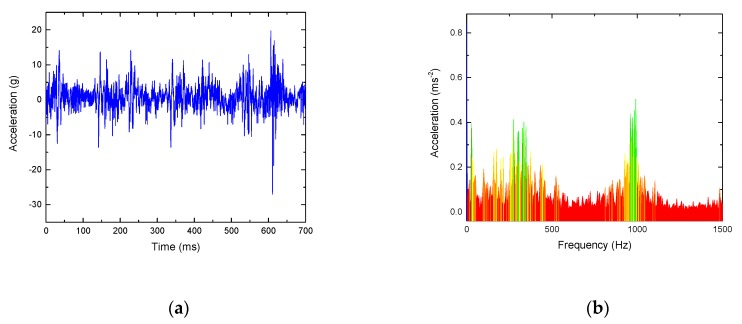
Spectrum analysis of a rail vibration signal with peeling damage. (**a**) Waveform; (**b**) frequency spectrum.

As shown in Figure 13, the signal waveform and frequency spectrum of a typical normal rail surface are mainly distributed in the low frequency part, usually less than 500 Hz. However, for the rail surface with peeling damage, the signal has an obvious distribution in the high frequency band, as shown in Figure 14, and the main frequency increases significantly at 1000 Hz. By calculation, its kurtosis value is 42.3 and approximate entropy is 0.332, which can effectively identify the rail faults.

- Corrugation damage

Undulating wear is the roughness of the rail surface along the longitudinal direction, which appears most often in mountain areas. The causes are complex, involving rolling contact mechanics, material friction, vehicle coupling dynamics, and vibration load, vibration frequency, material characteristics, foundation, etc.

**Figure 15:**
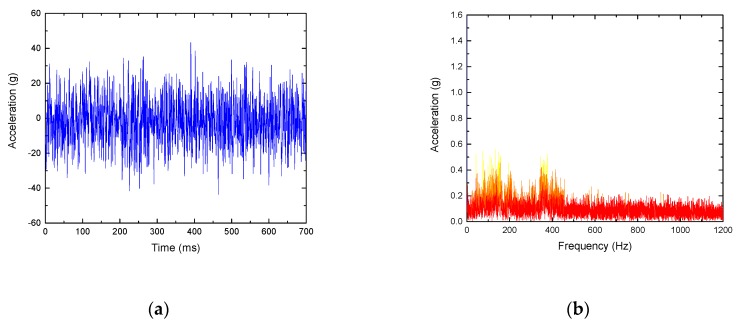
Analysis of a rail vibration signal with corrugation damage. (**a**) Waveform; (**b**) frequency spectrum.

As shown in Figure 15, the signal under undulating wear usually generates abnormal vibration in the frequency region corresponding to the middle frequency band and the undulating frequency band. Since the excitation frequency is the same as the natural frequency of the rail, resonance happens, so the signal has a large amount of energy. Its kurtosis value is 32.8 and approximate entropy is 0.735.

- Fault identification

**Figure 16:**
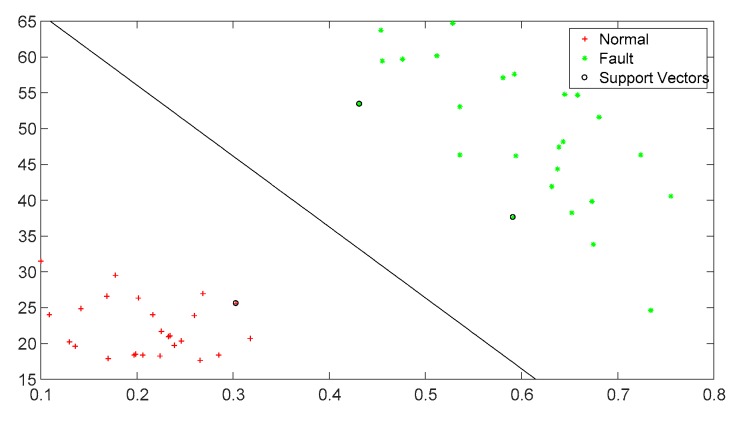
Classification result based on support vector machine (SVM) of the proposed method.

**Figure 17:**
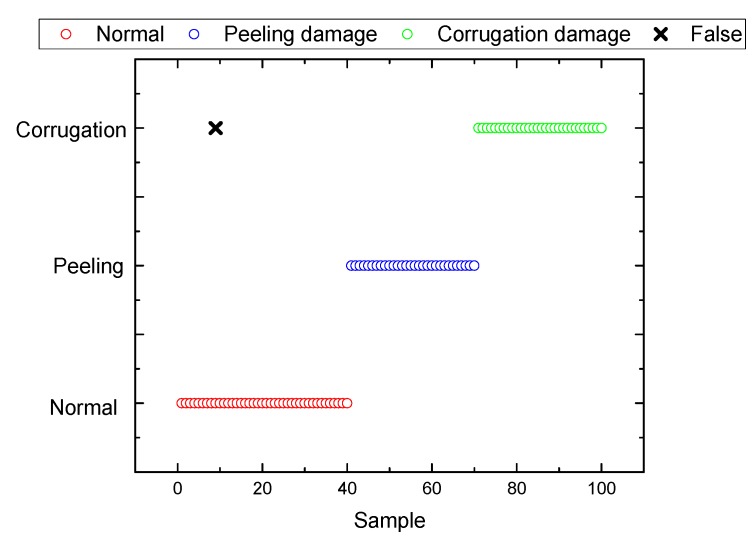
Identification accuracy of the proposed method.

**Figure 18:**
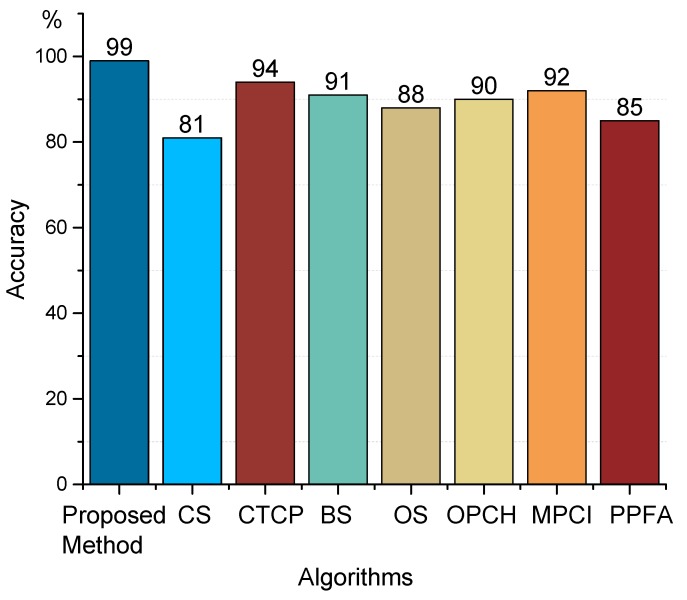
Identification accuracy of different algorithms.

A large number of experiments show that the fault signals’ kurtosis and approximate entropy calculated from corresponding reconstructed signals are both too large. The approximate entropy of fault signals is usually greater than 0.4 and the kurtosis is usually greater than 20. In this experiment, 100 sets of sample signals are used for identification and classification. The classification results based on the SVM and the fault identification accuracy of the proposed method in the paper are shown in Figure 16 and Figure 17, respectively. It can be seen that all signal types can be identified effectively except one, which is taking the corrugation damage fault as a normal one. The reason may be the influence of the rail surface roughness. When the kurtosis and approximate entropy calculated from original signals are directly used for identification and classification, there appear eight classification errors totally; the reason may be the influence of surface roughness. Therefore this kind of direct classification accuracy is very low. Besides, three classification errors appear with EMD algorithm based on the traditional cubic Hermite interpolation and the piecewise cubic Hermite interpolation. So it can be concluded that the method presented in this paper improves classification accuracy obviously. Figure 18 shows the accuracy of feature extraction and SVM recognition proposed in this paper by the eight methods mentioned in Table 1, which are CS (Cubic Spline), CTCP (Cubic Trigonometric Cardinal Precise spline [24]), BS (B-Spline [22]), OS (Direct constrained optimization [28]), OPCH (Optimized Piecewise Cubic Hermite [23]), MPCI (Monotone Piecewise Cubic Interpolation [29]), PPFA (Piecewise Power Function Algorithm [30]). By comparison, it is found that the comprehensive accuracy of this algorithm is superior to other algorithms.

## 6. Conclusions

Addressing the difficulties in rail compound fault diagnosis and the undershoot problem in vibration signal analysis based on the traditional EMD algorithms, an EMD algorithm based on the quartic C^2^ Hermite interpolation is presented in this paper. It replaces the traditional cubic Hermite interpolation algorithm and improves the performance of the EMD algorithm, and the undershoot problem in signal filtration based on the EMD algorithm is reduced effectively. As described, the principle components of the signal can be determined and reconstructed after SVD and cross correlation coefficient calculation. Then kurtosis and approximate entropy of the reconstructed signal are calculated as fault eigenvalues, which are used to classify the fault based on the SVM. Experimental results show that the EMD algorithm based on quartic C^2^ Hermite interpolation presented in this paper improves classification accuracy greatly.

## Figures and Tables

**Table 1:** Common interpolation methods and their advantages and disadvantages.

Interpolation Methods	Advantages	Disadvantages
Cubic Spline (CS)	Common algorithm,	Overshoot and undershoot,
CTCP [24]	good flexibility	high computational complexity
B-Spline (BS) [22]	Improve the local characteristics of EMD algorithm	The endpoint extension is assumed to be infinite, and the endpoint effect is not considered.
OS [28]	Adaptive and better than cubic spline	High computational complexity and poor smoothness.
OPCH [23]	Taking the difference between extremes as the cost function, the results are more accurate and reasonable than cubic spline	Slow computation speed and do not satisfy C^2^ continuous especially when there are many extreme points and cause undershooting.
MPCI [29]	Through strict theoretical analysis, it is proved that it can solve the overshoot problem caused by the cubic spline interpolation	Only satisfy C^1^ continuous, the curve smoothness is poor and undershoot.
Piecewise Power Function Algorithm (PPFA) [30]	It is more flexible than cubic spline interpolation, and the curve is smooth.	There is a contradiction between smoothness and flexibility and undershooting occurs.

**Table 2:** Calculation results.

Indicators	Cubic Hermite Interpolation Algorithm	Piecewise Cubic Hermite Interpolation Algorithm	Proposed Algorithm
IO	0.08899	0.05211	0.04573
IEC	0.3129	0.6534	0.9123

**Table 3:** Principal component of signal.

No.	Eigenvalues	Percentage of Current Eigenvalue in Total	Accumulated Percentage of the First K Eigenvalues in Total
1	29.2743	0.294605	0.294605
2	19.65641	0.197814	0.492419
3	12.7543	0.128354	0.620773
4	10.71655	0.107847	0.72862
5	5.604914	0.056406	0.785026
6	4.869944	0.049009	0.834035
7	4.58757	0.046167	0.880203
8	4.177541	0.042041	0.922244
9	3.021806	0.03041	0.952654
10	2.746282	0.027637	0.980292
11	1.95839	0.019708	1
